# Too Much and Too Little Medicine Are Two Sides of the Same Coin

**DOI:** 10.1111/ajd.14598

**Published:** 2025-09-08

**Authors:** Eugene Tan

**Affiliations:** ^1^ School of Clinical Medicine (St Vincent's Campus) UNSW Medicine Australia

**Keywords:** bias, eczema, error, evidence‐based medicine, isotretinoin, mental disorders, practice guidelines, selection bias, sexual dysfunction, steroids

Dear Editor,

The *Choosing Wisely* campaign highlights the harm of ‘Too Much Medicine’—overdiagnosis and unnecessary interventions. While much has been written about this topic, less attention has been paid to its counterpart—‘Too Little Medicine’. Two examples from the UK exemplify this; where effective treatments are restricted leading to undertreatment.

Isotretinoin has been linked to psychiatric and sexual side effects, resulting in usage restrictions [1]. Topical steroid withdrawal (TSW) is recognised as a distinct entity linked to prolonged use of topical steroids, prompting mandatory potency labelling and warnings [2]. Our college has issued statements contradicting these views. High‐quality studies do not support a causal link between isotretinoin and psychiatric or sexual dysfunction [3]. Similarly, topical steroids are safe and effective, but ‘steroid phobia’ triggers eczema flares in children [4]. These conflicting conclusions cannot both be correct.

Here, the COVID‐19 pandemic offers valuable insights. Policy responses varied widely—from strict lockdowns to minimal ones and from extensive use of drugs to minimal use of antivirals. What lessons can we draw in hindsight?

First, adhering to evidence‐based medicine (EBM) increases the probability of getting things right [5]. Numerous studies confirmed that even during a pandemic, rigorous clinical trials—including randomised controlled trials—are not only feasible but also essential [6]. Quality matters! More information does not mean more truth—of the quarter of a million articles on COVID‐19, many were of poor quality, and over 500 have been retracted [7].

Second, the GRADE framework ensures decisions consider benefits, harms, patient values, costs and feasibility [5]. For instance, mandated school closures affected 1.5 billion children globally, yet may not have been necessary to control viral spread [8]. Little attention was given to the negative effects—social isolation, mental health issues and lost education—which could have generational consequences [8].

It is concerning that policy makers did not grade the quality of evidence nor apply GRADE when evaluating isotretinoin or TSW [1, 2]. A myopic view of risk overlooks the harms of withholding effective treatment.

At best, isotretinoin restrictions will result in fewer patients receiving effective treatment. At worst, patients may suffer irreversible scarring and serious psychiatric illness, including depression and suicide. Similarly, the cautionary labelling of topical steroids may not improve eczema treatment but at worst, it may increase the risk of misdiagnosis. TSW is not a formally accepted diagnosis, with symptoms that overlap with many conditions. In the worst‐case scenario, misdiagnosing cutaneous T‐cell lymphoma—that can resemble eczema and is similarly unresponsive to topical steroids—for TSW could have serious, potentially life‐threatening consequences.

How can we mitigate this? We must demand that policymakers follow the framework of EBM and GRADE when making *any* public health decision [5]. Absolute knowledge is epistemologically unattainable, and mistakes are inevitable. However, by adhering to GRADE—systematically assessing the quality of evidence, weighing the relative importance of outcomes, balancing benefits against risks and integrating patient values, preferences and resource considerations—we can greatly reduce both the likelihood of errors and the impact of their consequences [5]. This systematic approach was absent in many public policies during the pandemic, raising concern that similar missteps may occur in the context of isotretinoin and topical steroids.

It is unreasonable to expect policymakers to have training in EBM, but not unreasonable to expect them to follow established frameworks, regardless of political pressure [9]. If EBM lies outside their expertise, they should engage with individuals who are trained in it. This framework ensures transparency, reduces confusion and improves public trust.

Too Much Medicine and Too Little Medicine are two sides of the same coin—both reflect a disregard for EBM. No society in history has ever suffered because its people demanded too much high‐quality evidence.

## Disclosure

Patient Consent for Publication: Not applicable.

Use of Artificial Intelligence (AI) Tools: The author declares that no generative AI or AI‐assisted technologies were used in the writing of this manuscript.

## Ethics Statement

The author has nothing to report.

## Conflicts of Interest

The author declares no conflicts of interest.

## Data Availability

Data sharing not applicable to this article as no datasets were generated or analysed during the current study.
